# Eco‐Friendly Synthesis of Selenium Nanoparticles via *Sternbergia candida*: Enhancing Antioxidant Defense and Mitigating Salt Stress in Pepper (*Capsicum annuum* L.) Plants

**DOI:** 10.1002/open.202400341

**Published:** 2025-01-28

**Authors:** Senem Kanber, Mahmut Yildiztekin, Mehmet Firat Baran

**Affiliations:** ^1^ Department of Environmental Sciences Institute of Science Muğla Sıtkı Koçman University 48000 Kötekli, Muğla Türkiye; ^2^ Department of Herbal and Animal Production Köyceğiz Vocational School 48800 Köyceğiz, Muğla Türkiye; ^3^ Department of Food Processing Vocational School of Technical Science 72060 Batman Türkiye

**Keywords:** Nanoparticle, Salt stress, *Capsicum annuum* L., Green synthesis, Selenium

## Abstract

Nanoparticles enhance agricultural applications with their bioactivity, bioavailability, and reactivity. Selenium mitigates the adverse effects of salinity on plant growth, boosting antioxidant defense, metabolism, and resilience to abiotic stress. Our study applied selenium nanoparticles to mitigate salinity‐induced damage and support plant growth. We characterized green‐synthesized nanoparticles and analyzed stress‐related metabolites, antioxidant activities (DPPH, ABTS), phenolic content, and reducing powers (CUPRAC, FRAP). Nanoparticle applications reduced proline and MDA levels while boosting chlorophyll, carotenoids, antioxidant activity (DPPH, ABTS), and total phenolic content. An increase was also observed in CUPRAC and FRAP reducing capacities. In terms of phenolic content, the highest value was determined in SA_1_ (4.58±0.40 mg GAE g^−1^) application; DPPH free radical scavenging activity IC50 value was determined in A_3_ (0.13±0.007 mg mL^−1^) application, which was closest to the positive control. The lowest proline level was found in A_3_ (15.00±0.64 nmol g^−1^ FW) and the lowest MDA level was found in SA_3_ (10.08±0.42 nmol g^−1^). Comparing the results, green synthesis of selenium nanoparticles using *Sternbergia candida* (SC‐SeNP) at different concentrations showed ameliorative effects on various parameters in plants, and it was determined that the effects of salt stress on pepper plants were reduced following SC‐SeNP applications.

## Graphical abstract

*Sternbergia candida* serves as a flexible reducing agent for the synthesis of SeNPs utilizing an economical and ecologically safe extract. Applications of selenium have demonstrated that plants cultivated in salinity preserve proper osmotic conditions and minimize cellular damage, as evidenced by photosynthetic pigments, proline, and MDA data. Because SeNPs are simple to use in agricultural activities, growers view them as crucial in terms of applicability.

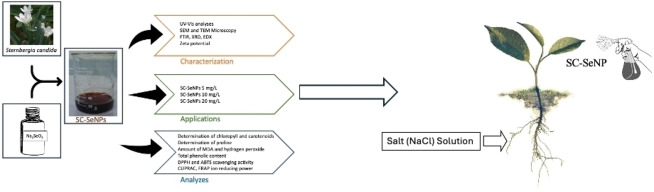

## 1 Introduction

Selenium (Se) is recognized as a beneficial element that enhances photosynthesis, secondary metabolites, and antioxidant metabolism in plant leaves. Many soils are considered deficient in selenium. To increase agricultural production, selenium applications are conducted either foliarly or through the soil to enrich plants with selenium. Through its synergistic and antagonistic relationships with different nutrients, selenium supports plant nutrition and plays a role in mitigating various abiotic stresses (drought, salinity, temperature, heavy metals). Selenium exists in nature in two forms: organic and inorganic. Selenate and selenite are the most common forms of selenium in soils. The primary soluble form of selenium in soil is selenate, which, due to its chemical similarity to sulfur, is transported from roots to leaves via sulfate carriers and is further synthesized in plastids.^[1]^

Foliar selenium applications result in the accumulation of selenium‐containing droplets on leaves, from where the element can enter the cells through various plant structures. Nutrients can easily move from areas of high concentration on the leaves to the inner parts through the pores present in the cuticles. The passage of Se depends on the size of the cuticles, the hydraulic conductivity of the cells, and the permeability of the stomata.^[2]^

Selenium contributes to antioxidant protection, improves metabolism, and regulates redox reactions under stress conditions. It also supports antioxidant enzyme activities, reduces oxidative stress biomarkers such as malondialdehyde, and decreases lipid peroxidation and thereby restoring plant growth, productivity, and crop quality under water deficiency stress.^[3]^

Soil salinity is one of the primary abiotic stresses affecting agricultural production. The FAO (Food and Agriculture Organization) report indicates that over 833 million hectares of soil and 10 % of agricultural land are affected by salt stress. Excessive salt accumulation in the soil occurs due to activities such as low rainfall, intensive fertilizer use, deforestation, and the erosion of saline rocks. This results in impaired stomatal conductivity, reduced photosynthetic efficiency, chlorosis, and leaf degradation, ultimately limiting plant growth and productivity due to the adverse effects on plant physiology.^[4]^

Pepper (*Capsicum annuum* L.) belongs to the Solanaceae family and can be cultivated in many regions of the world. Originating from South America, the cultivation of pepper dates back to ancient times. It was introduced to Spain from America in the 1400s. Besides adding color and flavor to dishes, its fruits are a rich source of minerals and vitamins.^[5]^ Due to its short growth period and high yield per unit area, it ranks high in cultivation.^[6]^ Salinity stress can cause reductions in yield and quality, leading to economic losses.

Nanoparticles contribute to agricultural applications due to their enhanced bioactivity, bioavailability, and reactivity, as well as their surface and adhesion effects. Nanofertilizers are macro or micronutrient fertilizers with sizes less than 100 nm used to increase crop productivity. They enhance nutrient use efficiency and reduce the amount of wasted nutrients by providing controlled and site‐specific delivery of nutrients. Traditional fertilizers have been reported to be actively utilized by plants in less than half of the applied nutrients.^[7]^

Green synthesis, or biological synthesis, refers to the synthesis of nanoparticles using biotechnological tools with the help of living organisms such as plants, microorganisms, and viruses. Green synthesis of nanoparticles involves the use of eco‐friendly, non‐toxic, and safe reagents.^[8]^

As a result of literature research, it was determined that different plants were used to synthesize selenium nanoparticles. Turna et al.^[9]^ reported that SeNPs synthesized from *Prunus armeniaca* L. leaf waste removed Congo red (CR) dye in aqueous solution. Zahidi et al.^[10]^ aimed to mitigate the effect of salinity on strawberry (*Fragaria* x *ananassa* Duch.) growth and yield by using SeNP via foliar spray method. When compared to groups that were not treated with nanoparticles, strawberry plants were shown to have improved growth and yield characteristics and to have higher levels of osmolytes, such as free proline and total soluble carbohydrate. Applying SeNP to the leaf has been shown to promote salinity tolerance by lowering stress‐induced lipid peroxidation and H_2_O_2_ (hydrogen peroxide) concentration. González‐García et al.^[11]^ reported that selenium, silicon and copper nanoparticles applied under salt stress increased chlorophyll and lycopene activity in the leaves of bell pepper (*Capsicum annuum* L.) fruits. In the study in which chitosan‐Selenium nanoparticles were applied as foliar spray in bitter melon (*Momordica charantia*), it was stated that salt stress decreased and growth and yield parameters increased. Depending on the nanoparticle application, proline concentration in plant tissues increased, MDA and H_2_O_2_ oxidants and Na aggregation decreased and salinity tolerance increased.^[12]^

*Sternbergia candida* Mathew et T. Baytop used for the green synthesis method, belonging to the Amaryllidaceae family, is a species that blooms white flowers in January and February. It is distributed in the Babadağ region of Fethiye, Muğla, Turkey. It contains Amaryllidaceous‐type alkaloids (belladin, lycorine, galanthamine, tazettine, etc.) known for their antiviral, immunostimulant, anticholinesterase, antileukemic, antitumor, and antimicrobial activities.^[13]^ They are rich in total phenolic content.^[14]^ When the literature was reviewed, it was observed that no study on the synthesis of selenium nanoparticles using *Sternbergia candida* plant was reported. Based on this, selenium nanoparticles were synthesized from *S. candida* (SC‐SeNP), which is known to have antioxidant activity and is expected to be biocompatible, and foliar spray applications were carried out on pepper plants at various concentrations.

In this study, SeNPs used at various concentrations were applied to pepper plants grown under salt stress, and the therapeutic effects of these nanoparticles on the stressed plants were investigated. Additionally, how the plant biochemistry changes was revealed with data. The aim of this study was to determine the optimum concentration of SeNPs that would highlight their beneficial effects on plants and serve as a foundation for future studies.

## Material and Methods

### Green Synthesis of SC‐SeNPs

*Sternbergia candida* (SC) Mathew et Baytop plants, collected from the Babadağ region of Fethiye, Muğla (Turkey). The plant diagnosis was carried out by Dr. Kenan AKBAŞ (Muğla Sıtkı Koçman University, Department of Biology); hence, their diagnosis was prepared following the herbarium and added to the collection. *S. candida* were first washed under tap water. They were then allowed to dry at room temperature after being repeatedly cleaned with distilled water. After being weighed, 100 g of the dried leaves were cooked in 250 mL of distilled water. *S. candida* extract were allowed to cool before being processed for nanoparticle synthesis by filtering them with Whatman 1.0 filter paper.^[15]^

**Figure 1:**
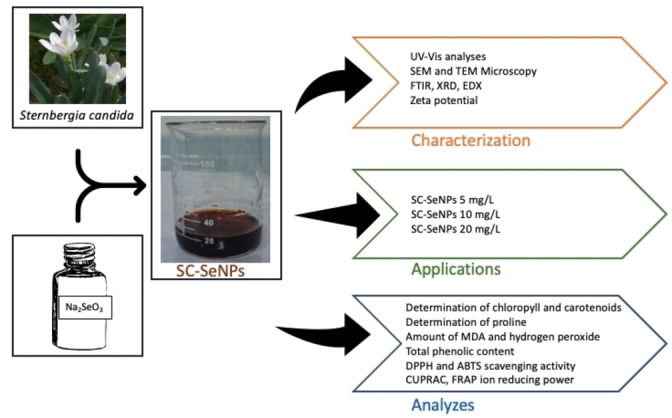
Steps of selenium nanoparticle synthesis derived from *Sternbergia candida*.

The synthesis of SC‐SeNPs was performed according to Alagesan and Venugopal.^[16]^ 100 mL of plant extract was mixed with 50 mM Sodium selenite (Na_2_SeO_3_) (Sigma Aldrich) salt, and the solution was allowed to react at 50 °C for one day (Figure 1). A color change in the mixture was observed and analyzed using UV‐Vis spectrophotometry. After the reaction was complete, the bio‐based SeNPs obtained were centrifuged at 6000 rpm. The solid part collected at the bottom of the tubes was washed several times with pure water to remove impurities. The solid part obtained was then placed in a glass petri dish and was then left to dry in an oven at 70 °C for 48 hours. The dried selenium nanoparticles were collected with a glass rod and prepared for use in biological applications. SC‐SeNPs were suspended in **phosphate buffered saline (PBS)** (pH 7.4) by ultrasonication and then centrifuged.

### Instrumentation

The synthesized SeNPs demonstrated maximum absorbance within the 290–800 nm wavelength range, measured using a spectrophotometer (Agilent CARY 60). The size, morphology, crystal structure, surface distribution, and zeta potential (ZP) of the SeNPs were analyzed using scanning electron microscopy (SEM) (EVO 40 LEQ), transmission electron microscopy (TEM) (JEOL JEM‐1010), energy dispersive X‐ray spectroscopy (EDX) (EVO 40 LEQ), X‐ray diffraction (XRD) (XRD D8 Discover), and a Zetasizer (Malvern Instruments Ltd.). The crystal size of SC‐SeNPs was calculated using the formula D=Kλ/(β cosθ) where D represents the size of the particle, K is constant value, λ is the X‐ray wavelength, β is the full‐width at half maximum, and θ is the Bragg angle.[^17^, ^18^] Additionally, Fourier transform infrared spectroscopy with attenuated total reflectance (FT‐IR ATR) (Shimadzu FTIR Spectrophotometer IRTracer‐100) was employed to identify the functional groups in the *Sternbergia candida* (SC) extract.

### Plant Material and Growth Conditions

In this study, the Vural F1 variety of pepper (*Capsicum annuum* L.), which is widely cultivated in the region and moderately resistant to salt stress, was utilized. The study was carried out as a pot experiment within a 500 square meter located in the Bodrum district of Muğla province. The pots were divided into plots for the treatments and arranged according to a randomized complete block design with three replications. A total of 24 pots were prepared, with 8 treatments and 3 replications each. Each 20 liter pot was filled with a 3 : 1 mixture of peat and perlite. Additionally, a 15 : 15 : 15 fertilizer was used in the study. Subsequently, two seedlings were planted in each pot, and the experiment was initiated with the first watering (2 L).

### Plant Treatments

In the study, 5, 10, and 20 mg L^−1^ selenium nanoparticles derived from *Sternbergia candida* (SC‐SeNPs) and a 100 mM NaCl solution to induce salt stress were applied to the pepper plants as indicated in the experimental design (Table 1).

**Table 1:** Experimental design.

1. C (Control) (A_0_)	5. S (Salt) (SA_0_)
2. SC‐SeNPs(1) (A_1_)	6. S+ SC‐SeNPs(1) (**SA_1_**)
3. SC‐SeNPs(2) (A_2_)	7. S+ SC‐SeNPs(2) (**SA_2_**)
4. SC‐SeNPs(3) (A_3_)	8. S+ SC‐SeNPs(3) (**SA_3_**)

A: Application C: Control Group, S: Salt (NaCl) (100 mM), SeNPs(1): Nanoparticle (5 mg/L), SeNPs(2): Nanoparticle (10 mg/L), SeNPs(3): Nanoparticle (20 mg/L), SC: *Sternbergia candida*

**Figure 2:**
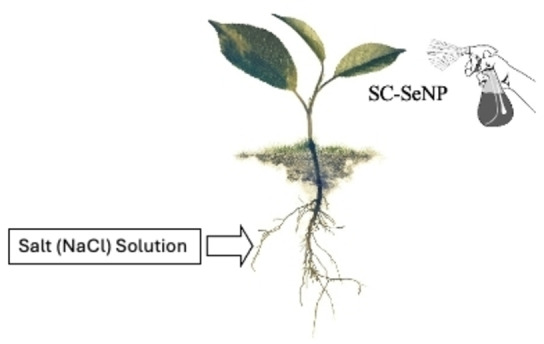
Application technique of nanoparticles (NP) and NaCl on pepper plants.

In the experiment, three different concentrations of Selenium nanoparticles (5, 10, and 20 mg L^−1^) were applied to pepper (*Capsicum annuum* L.) plants by foliar spray until all the leaves were completely wetted (Figure 2). The nanoparticle applications were performed to adapt the plant to the environment and were initiated at the beginning of the experiment, repeated every two weeks for a total of four times.

To induce salt stress in the plants, an application of 100 mM NaCl was carried out by mixing it with irrigation water. Each pot was watered three times a week with a total volume of 2 liters, with one of these waterings being saline water for the designated groups and plain irrigation water for the other groups.

### Physiological Activities

#### Determination of Chlorophyll and Carotenoids

Chlorophyll analysis was conducted by weighing 0.5 g of leaf samples from each group, which were then ground in a mortar with the addition of 80 % acetone containing 0.5 g CaCO_3_. After adding acetone to the mixture, it was centrifuged for 5 minutes. The supernatant was collected and subsequently treated with acetone. After preparation, the absorbance of the mixture was measured using a spectrophotometer (Rayleigh UV‐1601) at wavelengths of 450, 645, and 663 nm.^[19]^

#### Determination of Proline

The proline content in the leaves was determined according to Bates.^[20]^ Following homogenization with 3 % (w/v) sulfosalicylic acid, leaf samples from each group were filtered through Whatman no. 2 filter paper.

**Figure 3:**
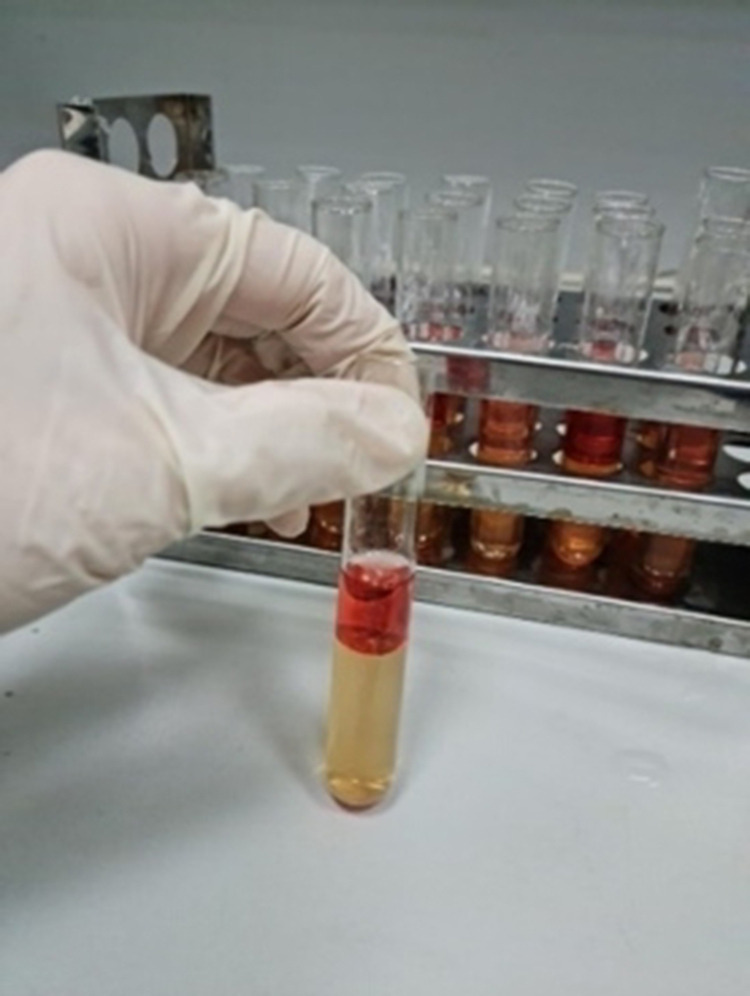
Proline analysis.

Glacial acetic acid and acid ninhydrin were added, and the mixture was kept in a water bath at 100 °C for 1 hour. The reaction was then stopped in an ice bath, and after the addition of toluene to the mixture (Figure 3), the fraction aspirated from the liquid phase was determined spectrophotometrically at 520 nm.

#### Determination of Lipid Peroxidation (MDA)

The level of the final product, malondialdehyde (MDA), was determined according to Madhava Rao and Sresty^[21]^ for the measurement of lipid peroxidation. Leaf samples weighing 0.5 g were homogenized with Tri‐chloroacetic acid (TCA), and the filtered samples were centrifuged at 10,000 rpm for 15 minutes. Following centrifugation, the supernatant was pipetted from the Eppendorf tubes, and a mixture of TBA (Thiobarbituric acid) and TCA was added. All experimental tubes were heated at 95 °C for 30 minutes. After removal from the water bath, the samples were placed in an ice bath, and their absorbance was measured at 532 and 600 nm.

### Antioxidant Activities

#### Preparation of Plant Extracts

The plants harvested at different times were dried in a cool, dark area. Once dried, they were pulverized using a blender, and 3 grams of the pulverized material was weighed on a precision balance and transferred to Erlenmeyer flasks. After adding 50 mL of solvent (methanol) and sealing the flasks, they were agitated in an orbital shaker incubator at 50 °C for 6 hours. Following this period, the samples were removed from the incubator, filtered through filter paper, and transferred into amber bottles. This process was repeated twice using the same procedure.

To remove the solvent (methanol) from the filtered extract solutions, a rotary evaporator (48 °C, 70 rpm) was used, followed by the use of a lyophilizer to remove any remaining water from the samples. The obtained extracts were stored at −20 °C for use in experiments.^[22]^

#### Quantification of Total Phenolic Content

The phosphomolybdate and phosphotungstate combination known as the Folin‐Ciocalteu reagent (sometimes called the Folin Phenol Reagent or Folin‐Denis reagent) is used in colorimetric analysis to measure antioxidants that are phenolic and polyphenolic. The analyses were modified according to the method described by Singleton et al.^[23]^ Gallic acid was used as a positive control in the experiment. To obtain a calibration curve for gallic acid, 1.8 mg of gallic acid was weighed to achieve a concentration of 1 mg mL^−1^, and the same amount was dissolved in methanol to make the concentration 1 mg mL^−1^. Five different concentrations (0.01 mg mL^−1^, 0.02 mg mL^−1^, 0.03 mg mL^−1^, 0.04 mg mL^−1^, 0.05 mg mL^−1^) were prepared to obtain the calibration curve for gallic acid. Phenolic compounds in the samples prepared from plant extracts dissolved in methanol at different concentrations reacted to form a colored complex with the Folin‐Ciocalteu reagent under alkaline conditions, and the maximum absorbance of the resulting purple color complex was measured at 700 nm in a spectrophotometer (Multiskan Go (Thermo)).

#### Determination of DPPH Radical Scavenging Activity

The determination of antioxidant activity of the samples was conducted according to Turan and Mammadov.^[24]^ This method relies on the spectrophotometric measurement of the reduction of the stable free radical 2,2‐diphenyl‐1‐picrylhydrazyl (DPPH) in the presence of antioxidant compounds that donate electrons and hydrogen atoms, resulting in the discoloration of the characteristic purple color.

Initially, 0.2 mL of 0.0004 % (w/v) methanolic DPPH solution was mixed with 0.05 mL (0.05–0.4 mg) of extract solutions. After incubating in the dark at room temperature for 30 minutes, the absorbance values were measured at 517 nm. Using the absorbance values of the samples;

The inhibition percentage was calculated using the formula:

$$\%\;inhibition=\;\frac{A\;control-A\;sample}{A\;control}X\;100$$

Using the obtained inhibition values, a graph was plotted against the concentrations of the extract determined in mg mL^−1^. The concentrations of the extracts that resulted in a 50 % color change (IC_50_ value) were calculated as the concentrations corresponding to 50 % inhibition. Butylated hydroxyanisole (BHA) was used as a positive control.

#### Determination of ABTS Radical Scavenging Activity

The ABTS free radical scavenging activity experiment was conducted according to the method described by Re et al.^[25]^ After preparing solutions of ABTS and potassium persulfate, they were mixed and left in the dark at room temperature for 12–14 hours. The mixture was then diluted with ethanol to an absorbance of 0.700 at 734 nm before the start of the experiment.

After that, 0.5 mL of the extract solution and 4.5 mL of the diluted mixture were added to the samples (0.05 mg mL^−1^, 0.1 mg mL^−1^, 0.15 mg mL^−1^, 0.2 mg mL^−1^, and 0.25 mg mL^−1^). Following mixing, the samples were left to incubate for half an hour at room temperature. A spectrophotometer was then used to measure the absorbance of the mixture at 734 nm. BHA was utilized as a positive control while ethanol served as a blind control. The following formula was used to get the inhibition percentage:

$$\%\;inhibition=\;\frac{A\;control-A\;sample}{A\;control}X\;100$$

The IC50 values were calculated based on the obtained inhibition percentages.

#### Activity of Cupric Ion Reducing (CUPRAC) Method

The method was performed to determine the copper (II) ion reduction activity (CUPRAC) according to Azmaz et al.^[26]^ To a pre‐mixed reaction mixture containing CuCl_2_ (17 mg, 10 mL), neocuproine (15 mg, 10 mL), and NH_4_Ac (0.7708 g, 10 mL) buffer, the extract solution was added. A buffer solution without extract was prepared as a blank. The absorbances of the combination and the blank were measured at 450 nm following a 30 minute incubation period at room temperature. The sample's absorbance was deducted from the blank's absorbance. Trolox equivalents, or mg TE/g of extract, were used to express CUPRAC activity.

#### Activity of Ferric Ion Reducing (FRAP) Method

The method was conducted to determine the ferric ion reduction activity (FRAP) according to Benzie and Strain.^[27]^ To 2 mL of FRAP reagent solution (3.6 pH, 0.3 M acetate buffer, 10 mM TPTZ, and 20 mM FeCl3), the extract solution dissolved in its own solvent at 1 mg/mL concentration was added. The absorbance of the samples was measured at 595 nm after incubation at room temperature for 30 minutes. Water was used as a blank. The obtained absorbance values were substituted into the trolox equivalent (mg TE g^−1^ extract) equation to calculate the results.

### Statistical Analysis

The study employed the SPSS (14.0) software package to perform a one‐way analysis of variance (ANOVA). The Least Significant Difference (LSD) test was used to determine the significance of any differences between groups that were found to be statistically significant (p <0.05). The data on antioxidant activity were evaluated using Minitab 16 software.

## 2 Results

### 2.1 Characterization of SC‐SeNPs

A dark reddish color was obtained as a result of the interaction between sodium selenite and *Sternbergia candida* (SC) extract during SeNP production. SC extract acted as a reducing agent in the reaction.

#### 2.1.1 UV‐Vis Spectrophotometric Characterization

**Figure 4:**
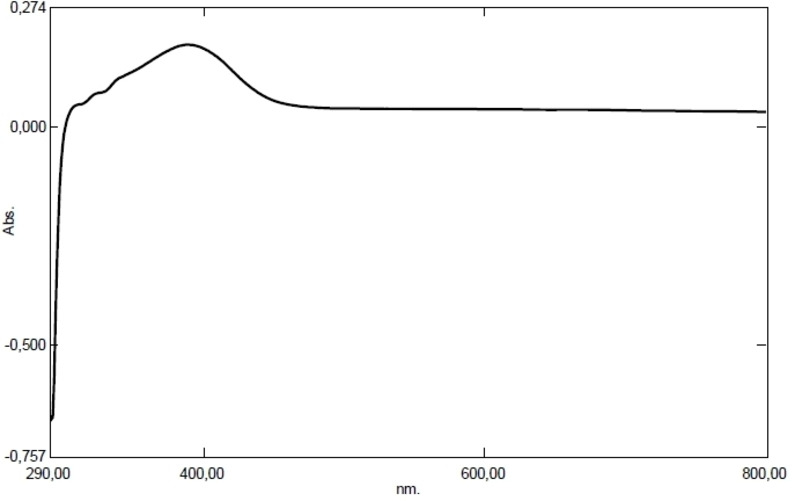
UV‐Vis diagram of SC‐SeNPs.

This finding was supported by the measurement of maximum absorbance at 388 nm in the UV‐Vis data of the samples taken from the reaction medium (Figure 4).

#### 2.1.2 X‐Ray Diffraction (XRD) Characterization of SC‐SeNPs

The phase angle and crystal structure of the SeNPs synthesized from the extract of the endemic plant *Sternbergia candida* were verified through an X‐ray diffraction (XRD) diagram. The crystallite size and phase of the synthesized SC‐SeNPs were assessed using the XRD pattern (Figure 3).

**Figure 5:**
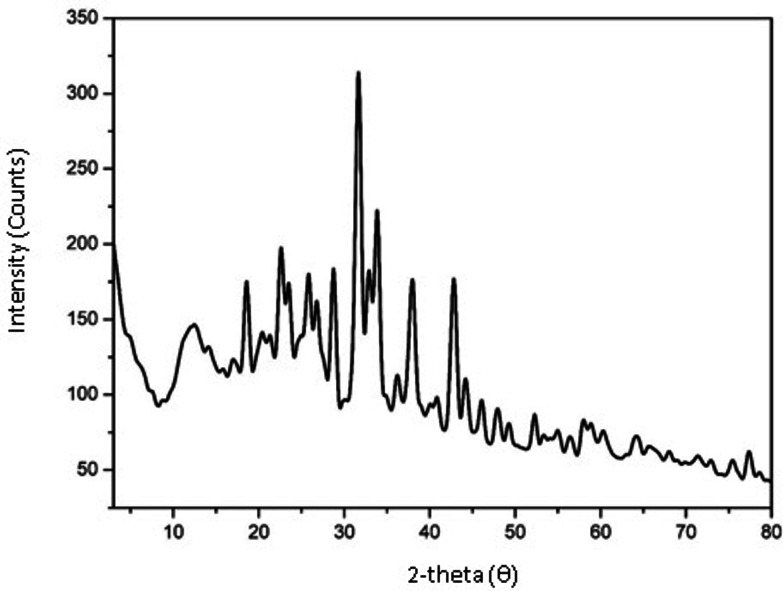
Crystal structures of the synthesized SC‐SeNPs reflected in the plane based on XRD data

In Figure 5, broadening at Bragg angles corresponding to selenium (100), (101), (110), (102), (200), and (210) planes was observed in the data collected at 2‐theta (θ). These points were measured at values of 23.50, 31.68, 37.60, 42.94, 52.34, and 77.40, respectively. The findings indicated that the crystal structures of the synthesized SC‐SeNPs were consistent with the Joint Committee on Powder Diffraction Standards (JCPDS file no. 65‐9729). According to the Debye‐Scherrer Equation (1), the crystal size was calculated to be 32.04 nm, based on the Bragg angle of the highest peak (31.68°) (FWHM: 0.450.

(1) $$D=k\lambda /\left(\beta cos\theta \right)$$

#### 2.1.3 FT‐IR Analysis of SC‐SeNPs

**Figure 6:**
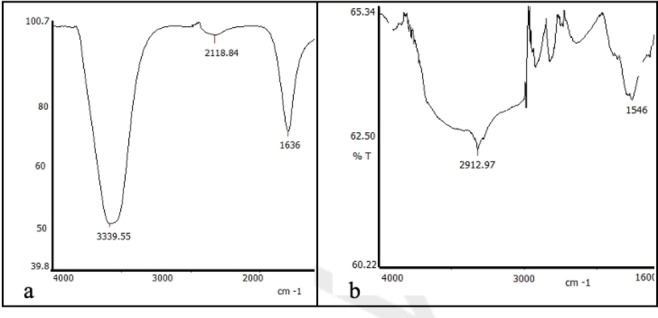
FT‐IR spectra of *Sternbergia candida* plant extract (a) and biosynthesized SeNPs (b)

The FTIR spectra of selenium nanoparticles reduced and synthesized using *Sternbergia candida* before and after the reaction are shown in Figures 6(a–b). Some peaks present in the aqueous extract before the reaction were identified as follows: the 3339.55 cm^−1^ peak corresponds to the −OH stretching band, the 2219 cm^−1^ peak corresponds to the −C−N triple bond, and the 1636 cm^−1^ peak corresponds to the −C=O carbonyl peak. The changes observed in the OH group and carbonyl groups after the reaction indicate that the reaction occurred on these functional groups. Additionally, the sharp peaks observed at 851 cm^−1^ and 665 cm^−1^ are thought to be associated with metallic bonds.

#### 2.1.4 Zeta Potential Analysis of SC‐SeNPs

**Figure 7:**
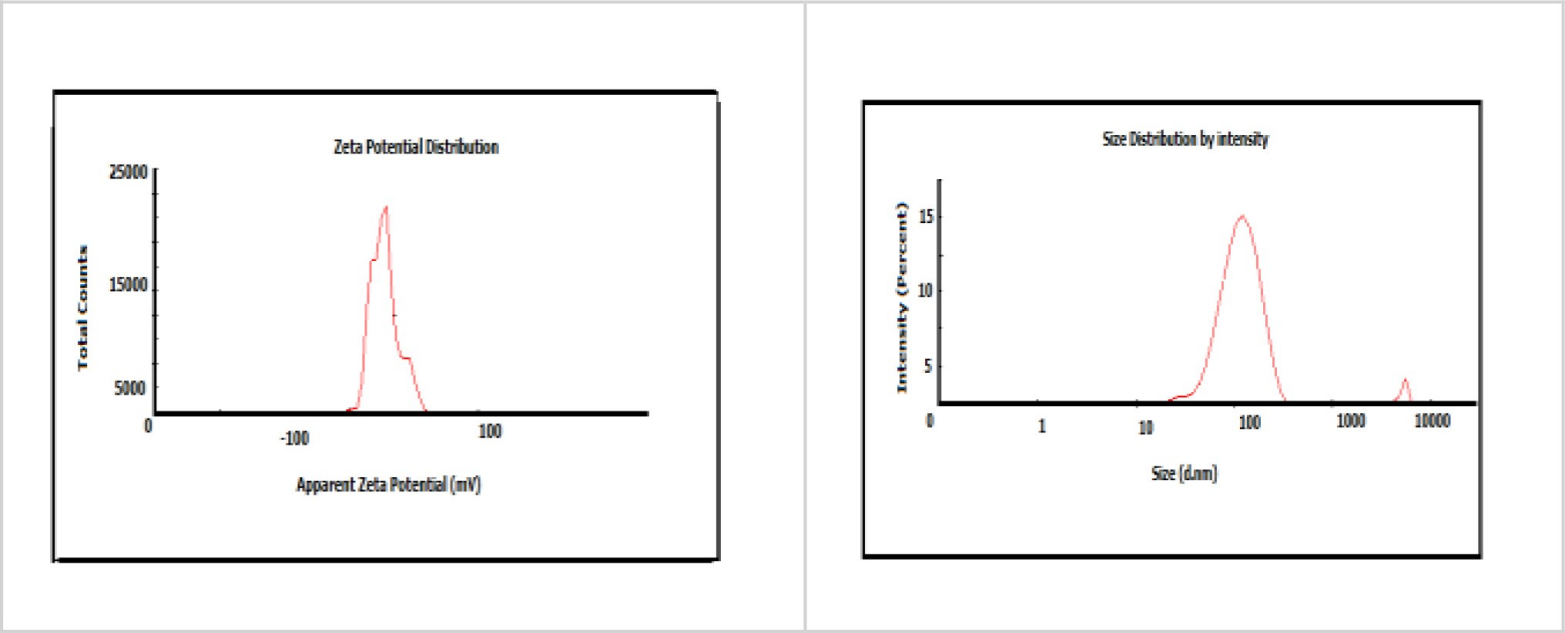
Zeta dimension and load distribution diagram of SC‐SeNPs

The negative charge distribution on the surface of biologically synthesized SC‐SeNPs is crucial for their stability and biocompatibility. Figure 7 shows that the SC‐SeNPs obtained in the study have a negative charge of −22.3 mV. SeNPs with negative surface charge distribution can readily interact with many biological structures that exhibit negative characteristics by displaying positive attributes in physiological environments.

#### 2.1.5 TEM, SEM and EDX Analysis of SC‐SeNPs

**Figure 8:**
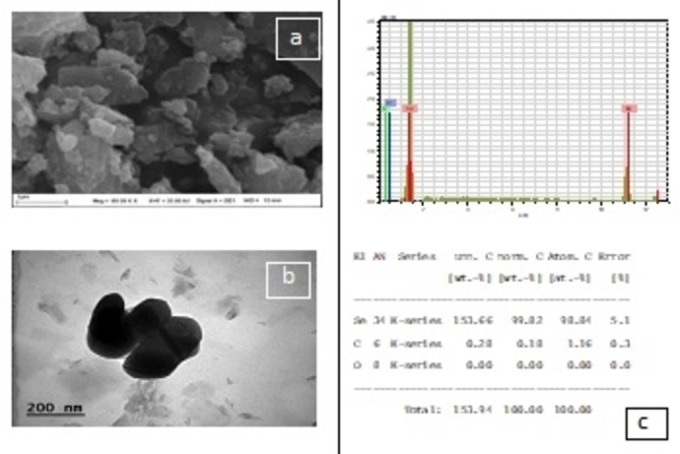
SEM (a), TEM (b) image and EDX spectrum data (c) of Se NPs produced from Sternbergia candida plant extract.

In Figure 8, selenium nanomaterials synthesized using *Sternbergia candida* extract were shown to form SeNPs due to the high presence of Se element in the elemental composition. The TEM, SEM, and EDX data provided in Figure 8 were used to determine the morphological characteristics of the SeNPs synthesized with *Sternbergia candida* extract. The images show that the SC‐SeNPs had a spherical morphology, with no evidence of clustering. When TEM images of SeNPs at 200 nm are examined, it is seen that they vary between 119.14–148.99 nm in average size.

### 2.2 Effects of SC‐SeNPs on the Physiological Activities

#### 2.2.1 Chloropyll and Carotenoid Contents

**Figure 9:**
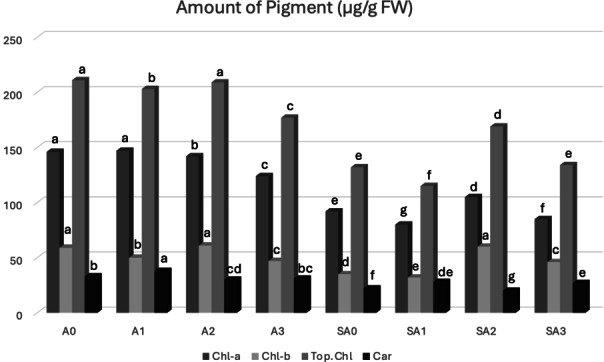
Chlorophyll and carotenoid levels (μg/g FW) of *Capsicum annuum* L. plant samples. SC: *Sternbergia candida*, SeNPs: Selenium nanoparticles.

Following selenium nanoparticle applications, the chlorophyll and carotenoid contents of *Capsicum annuum* L. leaves are presented in Figure 9.

A_0_: C (Control), A_1_: C+ SC‐SeNPs(1), A_2_: C+ SC‐SeNPs(2), A_3_: C+ SC‐SeNPs(3), SA_0_: S (Salt), SA_1_: S+ SC‐SeNPs(1), SA_2_: S+ SC‐SeNPs(2), SA_3_: S+ SC‐SeNPs(3), SeNPs(1): Nanoparticle (5 mg L^−1^), SeNPs(2): Nanoparticle (10 mg L^−1^), SeNPs(3): Nanoparticle (20 mg L^−1^). *Distinct letters indicate statistically significant differences (P≤0.05).

In our study, the highest content of chlorophyll a (Chl‐a) in *Capsicum annuum* L. plants was determined in the A_1_ treatment (146.87±2.33 μg g^−1^ FW) following SC‐SeNP applications. Decreases in chlorophyll levels were observed in the other treatments. The lowest Chl‐a content was observed in the SA_1_ treatment (79.82±0.45 μg g^−1^ FW). Among the treatment groups involved in the study, the selenium at the second concentration (10 mg L^−1^) was found to be more effective. Regarding chlorophyll b (Chl‐b) levels, the highest values were observed in the A_2_ (61.19±0.82 μg g^−1^ FW) and A_7_ (59.77±1.50 μg g^−1^ FW) treatments, while the lowest Chl‐b content was observed in the SA_1_ treatment (31.92±1.26 μg g^−1^ FW). When examining carotenoid levels, the highest values were determined in the A_1_ (38.00±0.49 μg g^−1^ FW) and SA_1_ (27.67±1.04 μg g^−1^ FW) treatments, while the lowest value was detected in the SA_2_ treatment (19.58±0.86 μg g^−1^ FW) with nanoparticle application.

#### 2.2.2 Proline Contents

The proline content of the plants following selenium nanoparticle applications is presented in Table 2.

**Table 2:** Proline content (nmol g^−1^ FW) of *Capsicum annuum* L. plant samples.

TREATMENTS	Prolin (nmol g^−1^ FW)
A_0_	28.23±0.53^[c]^
A_1_	21.40±0.39^[e]^
A_2_	17.64±0.92^[g]^
A_3_	15.00±0.64^[h]^
SA_0_	34.46±0.39^[a]^
SA_1_	30.19±0.77^[b]^
SA_2_	23.11±0.64^[c]^
SA_3_	19.09±0.78^[c]^

* Values are shown as mean±standard deviation; there is no statistically significant difference (p<0.05) between means within the same column that are followed by the same letter. SC: *Sternbergia candida*, SeNPs: Selenium nanoparticles. A_0_: C (Control), A_1_: C+ SC‐SeNPs(1), A_2_: C+ SC‐SeNPs(2), A_3_: C+ SC‐SeNPs(3), SA_0_: S (Salt), SA_1_: S+ SC‐SeNPs(1), SA_2_: S+ SC‐SeNPs(2), SA_3_: S+ SC‐SeNPs(3), SeNPs(1): Nanoparticle (5 mg/L), SeNPs(2): Nanoparticle (10 mg mL^−1^), SeNPs(3): Nanoparticle (20 mg mL^−1^).

When comparing the proline content after SC‐SeNPs applications with the control groups, the highest values were observed in the A_1_ (21.40±0.39 nmol g^−1^ FW) and SA_1_ (30.19±0.77 nmol g^−1^ FW) treatments. The lowest proline level was found in the A_3_ treatment (15.00±0.64 nmol g^−1^ FW). Salt treatment increased the proline level in the control group by 22.07 %, while the proline level decreased by 44.60 % following the SA_3_ treatment.

#### 2.2.3 Lipid Peroxidation (MDA) Contents

The lipid peroxidation (MDA) levels determined after nanoparticle applications at various concentrations are presented in Table 3.

**Table 3:** Lipid peroxidation (MDA) levels (nmol/g) in *Capsicum annuum* L. plant samples.

TREATMENTS	MDA (nmol g^−1^)
A_0_	8.48±0.29^[d]^
A_1_	7.57±0.12^[e]^
A_2_	4.48±0.29^[f]^
A_3_	7.57±0.24^[e]^
SA_0_	31.28±0.70^[a]^
SA_1_	14.37±0.44^[b]^
SA_2_	10.43±0.32^[c]^
SA_3_	10.08±0.42^[e]^

* Values are shown as mean±standard deviation; there is no statistically significant difference (p<0.05) between means within the same column that are followed by the same letter. SC: *Sternbergia candida*, SeNPs: Selenium nanoparticles. A_0_: C (Control), A_1_: C+ SC‐SeNPs(1), A_2_: C+ SC‐SeNPs(2), A_3_: C+ SC‐SeNPs(3), SA_0_: S (Salt), SA_1_: S+ SC‐SeNPs(1), SA_2_: S+ SC‐SeNPs(2), SA_3_: S+ SC‐SeNPs(3), SeNPs(1): Nanoparticle (5 mg L^−1^), SeNPs(2): Nanoparticle (10 mg L^−1^), SeNPs(3): Nanoparticle (20 mg L^−1^).

As a result of the SC‐SeNPs applications, the highest MDA levels were observed in the A_1_ (7.57±0.12 nmol g^−1^), A_3_ (7.57±0.24 nmol/g), and SA_1_ (14.37±0.44 nmol g^−1^) treatments. The most significant decreases were detected in the SA_2_ (10.43±0.32 nmol g^−1^) and SA_3_ (10.08±0.42 nmol g^−1^) treatments.

### 2.3 Effects of SC‐SeNPs on the Antioxidant Activities

#### 2.3.1 Quantification of Total Phenolic Content

Table 4 displays the total phenolic content of the extracts made from *Capsicum annuum* L. that were determined by the Folin‐Ciocalteu method. The total phenolic content was calculated as gallic acid equivalent (mg GAE/g) using the equation derived from the calibration curve drawn with four different concentrations of gallic acid.

**Table 4:** Total phenolic content (mg GAE g^−1^) of *Capsicum annuum* L. plant extracts.

TREATMENTS	Total Phenolic Content
A_0_	3.60±0.20^[a,b]^
A_1_	4.54±0.47^[a,b]^
A_2_	3.58±0.06^[a,b]^
A_3_	3.78±0.21^[a,b]^
SA_0_	3.11±0.33^[b]^
SA_1_	4.58±0.40^[a]^
SA_2_	2.78±0.20^[b]^
SA_3_	3.45±0.20^[a,b]^

*Values are shown as mean±standard deviation; means within the same column separated by the same letter are not statistically significantly different (p<0.05). GAE/g: Gallic Acid Equivalent per gram. SC: *Sternbergia candida*, SeNPs: Selenium nanoparticles. A0: C (Control), A1: C+ SC‐SeNPs(1), A2: C+ SC‐SeNPs(2), A3: C+ SC‐SeNPs(3), SA0: S (Salt), SA1: S+ SC‐SeNPs(1), SA2: S+ SC‐SeNPs(2), SA3: S+ SC‐SeNPs(3). SeNPs(1): Nanoparticle (5 mg L^−1^), SeNPs(2): Nanoparticle (10 mg L^−1^), SeNPs(3): Nanoparticle (20 mg L^−1^).

When compared to the salt application (SA_0_), the highest phenolic value was determined to be 4.58±0.40 mg GAE g^−1^ in the SA_1_ treatment. It was found that the group with the lowest phenolic content had a value of 2.78±0.20 mg GAE g^−1^ in the SA_2_ treatment.

#### 2.3.2 Radical Scavenging Activity

*Capsicum annuum* L. plant methanol extracts were subjected to two different antioxidant analyses, namely DPPH and ABTS. The results of these analyses are presented in Table 5.

**Table 5:** DPPH and ABTS radical scavenging activities (mg mL^−1^, IC_50_) of *Capsicum annuum* L. extracts.

TREATMENTS	DPPH	ABTS
A_0_	0.07±0.014^[d]^	0,12±0,008^[c]^
A_1_	0.17±0.011^[a,b,c]^	0,07±0,002^[d]^
A_2_	0.18±0.008^[a,b]^	0,07±0,003^[d]^
A_3_	0.13±0.007^[c]^	0,06±0,004^[d,e]^
SA_0_	0.15±0.004^[b,c]^	0,25±0,003^[a]^
SA_1_	0.18±0.003^[a,b]^	0,14±0,002^[b]^
SA_2_	0.20±0.005^[a]^	0,06±0,006^[d,e]^
SA_3_	0.16±0.017^[b,c]^	0,05±0,003^[e]^
BHA	0.02±0.0002^[e]^	0,02±0,0004^[f]^

*Values are shown as mean±standard deviation; means within the same column separated by the same letter are not statistically significantly different (p<0.05). BHA: Butylated Hydroxyanisole, IC_50_: Half‐maximal inhibitory concentration. SC: *Sternbergia candida*, SeNPs: Selenium nanoparticles. A0: C (Control), A1: C+ SC‐SeNPs(1), A2: C+ SC‐SeNPs(2), A3: C+ SC‐SeNPs(3), SA0: S (Salt), SA1: S+ SC‐SeNPs(1), SA2: S+ SC‐SeNPs(2), SA3: S+ SC‐SeNPs(3). SeNPs(1): Nanoparticle (5 mg L^−1^), SeNPs(2): Nanoparticle (10 mg L^−1^), SeNPs(3): Nanoparticle (20 mg L^−1^).

DPPH and ABTS radicals are known and can be scavenged by antioxidants.^[19]^ According to the values given in Table 5 for the DPPH assay, the A_3_ (0.13±0.007 mg mL^−1^, IC_50_) and SA_3_ (0.16±0.017 mg mL^−1^, IC_50_) applications exhibited the highest DPPH radical scavenging activities among the *Capsicum annuum* L. plant extracts. The lowest DPPH radical scavenging activity was determined in the SA_2_ (0.20±0.005 mg mL^−1^, IC_50_) application. The best activity value determined in terms of DPPH radical scavenging activity is quite low compared to the synthetic antioxidant BHA (0.02±0.0002 mg mL^−1^, IC_50_). The best DPPH radical scavenging activity in both application groups was detected in SC‐SeNPs(3) extracts.

In the ABTS assay, compared to the BHA standard (0.02±0.0004 mg mL^−1^, IC_50_), the highest radical scavenging activity was observed in the A_3_ (0.06±0.004 mg mL^−1^, IC_50_) and SA_3_ (0.05±0.003 mg mL^−1^, IC_50_) applications, while the lowest ABTS radical scavenging activity was observed in the A_1_ (0.07±0.002 mg mL^−1^, IC_50_) and A_2_ (0.07±0.003 mg mL^−1^, IC_50_) applications, similarly to the salt application, which was observed in the SA_1_ (0.14±0.002 mg mL^−1^, IC_50_) application.

#### 2.3.3 Reducing Power

Table 6 presents the results of the FRAP and CUPRAC reducing power activity assays conducted with methanol extracts of *Capsicum annuum* L. plant.

**Table 6:** The reduction power activities of *Capsicum annuum* L. plant extracts measured by CUPRAC and FRAP assays (1.0 mg mL^−1^ (mg TE/g extract)±standard error).

TREATMENTS	CUPRAC	FRAP
A_0_	11,5±0,07^[f]^	1,63±0,02^[f]^
A_1_	15,1±0,21^[e]^	2,27±0,08^[d,e]^
A_2_	18,7±0,14^[c]^	2,50±0,06^[c,d]^
A_3_	20,3±0,10^[c]^	2,60±0,12^[c]^
SA_0_	16,8±0,08^[d]^	2,14±0,07^[e]^
SA_1_	27,69±0,85^[b]^	3,47±0,12^[b]^
SA_2_	28,48±0,73^[b]^	3,30±0,11^[b]^
SA_3_	34,27±0,53^[a]^	3,89±0,07^[a]^

*Values are shown as mean±standard deviation; means within the same column separated by the same letter are not statistically significantly different (p<0.05). TE g^−1^: Trolox Equivalent per gram. SC: *Sternbergia candida*, SeNPs: Selenium nanoparticles A0: C (Control), A1: C+ SC‐SeNPs(1), A2: C+ SC‐SeNPs(2), A3: C+ SC‐SeNPs(3), SA0: S (Salt), SA1: S+ SC‐SeNPs(1), SA2: S+ SC‐SeNPs(2), SA3: S+ SC‐SeNPs(3). SeNPs(1): Nanoparticle (5 mg L^−1^), SeNPs(2): Nanoparticle (10 mg L^−1^), SeNPs(3): Nanoparticle (20 mg L^−1^).

In the context of reducing power assays, the results of the CUPRAC (Cupric Ion Reducing Antioxidant Capacity) experiment indicated that the highest reducing power was observed in the SA_3_ group with a value of 34.27±0.53 mg TE g^−1^ extract, followed by the A_3_ group with a value of 20.3±0.10 mg TE g^−1^ extract. Conversely, the lowest reducing power was recorded in the A_1_ group with a value of 15.1±0.21 mg TE g^−1^ extract and in the SA_1_ group with a value of 27.69±0.85 mg TE g^−1^ extract.

Regarding the FRAP results, the highest reducing power was observed in the A_3_ group with a value of 2.60±0.12 mg TE g^−1^ extract and in the SA_3_ group with a value of 3.89±0.07 mg TE g^−1^ extract. Conversely, the lowest reducing power was detected in the A_1_ group with a value of 2.27±0.08 mg TE g^−1^ extract and in the SA_2_ group with a value of 3.30±0.11 mg TE g^−1^ extract.

## 3 Discussion

The use of plant materials is very valuable in terms of sustainability, environmentally friendly and non‐toxic. Although nanoparticle synthesis studies with herbal ingredients have increased in recent years, no nanoparticle synthesized from *Sternbergia candida* have been reported and this shows the originality of our study. The interaction of sodium selenite with *Sternbergia candida* (SC) extract in the synthesis of SeNP resulted in a dark red color. SC extract acted as the reaction's reducing agent. The fact that SeNPs obtained from citrus fruits reveal a similar color confirms our study.^[28]^ According to the UV‐Vis spectroscopy results, SC‐SeNPs exhibited maximum absorbance at 388 nm. Similarly, SeNPs synthesized using *Urtica dioica* demonstrated maximum absorbance at 376 nm.^[29]^ Similar results to the XRD pattern (Figure 5) of SC‐SeNPs synthesized in our study were observed in selenium nanoparticles synthesized by Olaoye et al.^[30]^ using *Moringa oleifera* leaf and bark extracts. The 2‐theta (θ) values of the nanoparticles synthesized from the peel were reported as 20.5°, 22.54°, 24.56°, 26.3°, 31.9°, 32.78°, 36.94°, 37.86°, 45.2°, 55.4 and 68.3°, respectively. Similar results were obtained in SeNPs synthesized using orange peel waste.^[31]^ The crystal size of CF‐SeNPs synthesized by Solmaz et al.^[32]^ using agricultural *Citrus fortunella* (CF) wastes was found to be 27.58 nm, close to our study. FTIR analysis results reveal the bonds and functional groups responsible for the synthesis of nanoparticles and the stability of the synthesized nanoparticles. The FTIR analysis diagrams of SC‐SeNPs (Figure 6a–b) highlight the functional groups involved in the reaction. The analysis results of SeNPs synthesized from *Nyctantes arbor‐tristis* L. showed an absorption peak at approximately 3219 cm^−1^ due to the O−H bonding of alcohols and phenols.^[33]^ The data obtained in our study are also supported by the green synthesis performed with *Ceropegia bulbosa* extract.^[34]^ The stability of the colloidal solution of the synthesized SC‐SeNPs was determined by zeta potential analysis. The results of the zeta potential analysis are consistent with those reported by other researchers. Zeta potential analysis reported that Se‐NPs synthesized using *Rosmarinus officinalis* extract had a negative charge (−78 mV).^[35]^ Additionally, zeta potential analysis indicated that Se‐NPs synthesized using *Asteriscus graveolens* extract had a negative charge (−24.1 mV), which provides high physical stability. The negative surface charge also prevents conditions that may lead to instability, such as fluctuations and aggregation.^[36]^ In TEM, SEM and EDX analyses performed with the synthesized SC‐SeNPs, the nanoparticles showed a spherical morphology (Figure 8). The low signals for elements such as oxygen and carbon in the graph indicate the bioactive components present in the phytochemicals of the extract. These secondary metabolites play a role in coating and stabilizing the SC‐SeNPs. TEM image of selenium nanoparticles synthesized from *Artemisia chamaemelifolia* revealed nanoparticles with a size of approximately 110 nm. SEM images also revealed spherical morphology. Similar to our study, energy dispersive X‐ray (EDX) spectroscopy confirmed the presence of selenium (Se), carbon (C) and oxygen (O) elements in the sample. These elements indicate the presence of organic molecules on the surface of SeNPs, which act as a covering agent.^[37]^

Plants exposed to environmental stress suffer damage to chloroplasts, resulting in a decrease in photosynthesis. The addition of selenium in appropriate amounts can mitigate chloroplast damage and increase chlorophyll and carotenoid content.^[1]^ The lowest chlorophyll a (Chl‐a) content was observed in the SA_1_ application, showing a decrease of 13.67 % compared to salt application. Additionally, the Chl‐a content increased by 13.88 % compared to the control group following salt application. Selenium applications in salt‐stressed plants demonstrated that the second concentration of selenium was the most effective in increasing the Chl‐b content, with a 68.93 % increase. The lowest Chl‐b content was recorded in the SA_1_ application, with a decrease of 9.78 %. Salt application caused a 37.50 % reduction in the total chlorophyll content. The highest total chlorophyll levels were observed in the A_3_ and SA_3_ applications, whereas the lowest total chlorophyll content was found in the SA_2_ application. When the experimental groups were evaluated together, it was determined that the total chlorophyll content increased by 27.71 % after salt application. Despite the adverse effects of salt stress on plant chlorophyll levels, SC‐SeNP(2) applications were found to have a healing effect. Salt application caused a 33.43 % reduction in the carotenoid content of the plants. The carotenoid analysis revealed that the highest content was observed in the SC‐SeNP(1) concentration, with a 24.41 % increase in carotenoid levels in salt‐stressed pepper plants. In a study investigating the effects of leaf‐applied Chitosan‐Selenium nanoparticles (Cs−Se NP) on reducing salt stress in bitter melon (*Momordica charantia*), similar reductions in chlorophyll a, chlorophyll b, total chlorophyll, and carotenoid levels due to salt stress were reported. Compared to control plants treated with NP spray alone, the leaf application of 20 mg L^−1^ Cs−Se NP increased Chl‐a by 9.88 %, Chl‐b by 8.35 %, total chlorophyll by 9.40 %, and carotenoid content by 6.58 %.^[38]^ González‐Garcia et al.^[39]^ investigated the effects of selenium nanoparticles at various concentrations on the bioactive compounds of pepper (*Capsicum annuum* L.) fruits under salinity stress. They reported that the application of 50 mg L^−1^ SeNP reduced Chl‐a content by 24 % compared to the control in unstressed plants.

Proline, an osmotic protector, plays a role in scavenging free radicals and maintaining subcellular structures. It acts as an osmolyte, scavenging free radicals generated in plants due to stress, ensuring protein stability, and balancing stress factors.^[40]^ Compared to the SC‐SeNP application groups, the highest values were recorded with a decrease of 24.19 % in the A1 and 12.39 % in the SA1 applications. The lowest proline level observed in the A3 application can be attributed to the lack of exposure to salt stress in the experiment. A consistent decrease in proline levels was observed with increasing selenium concentration applied in all experiments. The data indicate that as the concentration of SC‐SeNP increases, stress in the plants decreases (Table 2). Ghasemian et al.^[41]^ reported that both salt stress and SeNP usage significantly altered proline concentrations in *Melissa officinalis* plants in their study aimed at alleviating salt stress with SeNPs. They reported the highest proline content as 0.123 μmol g^−1^ FW in plants exposed to 150 mM NaCl and 100 mg L^−1^ SeNP. Additionally, it was noted that in plants treated with 50 mg L^−1^ SeNP, proline content could resist changes under NaCl conditions ranging from 0 to 150 mM. In a study focused on the reduction of salinity stress in early seedling stages of canola (*Brassica napus* L.) productivity through exogenous applications of bio‐selenium nanoparticles, it was reported that under salinity stress conditions, non‐primed seedlings with SeNPs accumulated proline content by 149.8 % (Yanyou 9) and 99.59 % (Zhongshuang 11) compared to control group seedlings. It was further reported that seed priming with SeNPs significantly reduced proline accumulation under salinity stress during early growth stages, decreasing proline levels by 19.70 % and 38.37 % in Yanyou 9 and Zhongshuang 11, respectively, compared to non‐primed NaCl seedlings, especially at the highest SeNP concentrations (150 μmol L^−1^).^[42]^

Stress conditions in plants lead to the generation of reactive oxygen species, which oxidize lipids in plant cell membranes, causing structural disruptions. The selective permeability of the damaged membrane decreases or disappears. Malondialdehyde (MDA) emerges as a byproduct of lipid oxidation in cell membranes and serves as an indicator of damage severity in salt stress studies.^[43]^ In our study, salt stress caused a 268.87 % increase in the plant's MDA content. Among the experimental groups, the SC‐SeNP(2) concentration was found to be the most effective. The SC‐SeNP(2) application under salt stress reduced MDA levels by approximately 66.66 %. The data obtained in our study are supported by studies on the effects of salinity and selenium nanoparticles on MDA content in *Ocimum basilicum* L. seedlings, where a single SeNP application resulted in a 10 % decrease in MDA content compared to the control. When SeNPs were applied alongside 50 mM and 100 mM NaCl concentrations and compared with single applications, decreases in MDA content similar to our study were observed.^[44]^ Zahedi et al.^[45]^ investigated the effects of different salinity levels on the growth and yield of strawberry (*Fragaria ×ananassa* Duch.) plants using foliar spray applications of selenium nanoparticles. It was reported that, compared to the control group without salt application, 25 mM NaCl conditions increased MDA levels by 71.11 % and 20.33 % in the studies conducted in 2017 and 2018, respectively. Under 75 mM NaCl conditions, the increases in MDA levels were reported as 126.22 % and 37.88 %, respectively. Conversely, at 75 mM NaCl, applying 20 mg L^−1^ SeNPs reduced MDA levels by 19.64 % in 2017 and 5.45 % in 2018.

The highest phenolic content was observed in the SA_1_ application, showing a 47.27 % increase compared to salt treatments (SA_0_). The lowest value was recorded in the SA_2_ application, with a 10.61 % decrease. In *Capsicum annuum* L. plants, the phenolic content of the control group decreased by 13.61 % following salt application. Among the nanoparticle applications, the highest total phenolic content was observed in SC‐SeNPs(1) across both experimental groups. Sarkar and Kalita^[46]^ conducted a study on mustard plants under salt stress, using green synthesis with *Vitis vinifera* L. (GE) aqueous extract, and reported that a concentration of 30 mg L^−1^ GESeNP increased the plant's phenolic content by 98.88 %. Sheikhalipour et al.^[38]^ investigated the alleviating effects of foliar applications of chitosan‐selenium (Cs−Se) nanoparticles on *Stevia rebaudiana* Bertoni plants under salt stress. They reported that 20 mg L^−1^ Cs−Se NP application at 50 mM and 100 mM salinity levels increased total phenolic content by 3.24 % and 5.07 %, respectively, compared to the untreated group. A study evaluating fruit yield and quality in pomegranate plants (*Punica granatum* cv. Malase Saveh) after foliar application of nano‐selenium was conducted in 2016 and 2017. In 2016, it was observed that applying 2 μM SeNP (406.67±0.36 mg GAE/100 g fruit juice) increased phenolic content compared to 1 μM SeNP (393.00±0.25 mg GAE/100 g fruit juice). Similarly, in 2017, applying 2 μM SeNP (411.33±0.54 mg GAE/100 g fruit juice) increased phenolic content compared to 1 μM SeNP (394.00±0.47 mg GAE/100 g fruit juice).^[47]^

The DPPH scavenging activity assay evaluates the capacity of antioxidants to neutralize the stable organic nitrogen radical DPPH. The solution's color fades upon the addition of antioxidants, indicating their presence. Salt application decreased the DPPH scavenging activity in the control group by 114.28 %. The lowest DPPH scavenging activity was recorded with a 33.33 % decrease in the SA_2_ application. An initial decrease in DPPH scavenging activity was observed as the SeNP concentration increased, followed by a subsequent rise. Similar studies exist in the literature. For instance, a study on the effects of green‐synthesized SeNPs on lemongrass (*Cymbopogon* spp.) reported that applying 40 mg L^−1^ SeNP among various nanoparticle concentrations enhanced the plant's DPPH antioxidant activity (0.92±0.03 IC50). The IC_50_ value for the 20 mg L^−1^ SeNP application (1.61±0.02) indicated lower scavenging activity compared to our study (0.13±0.007).^[48]^ A study by González‐Lemus et al.^[49]^ investigated the nutritional parameters, biomass production, and antioxidant activity of *Festuca arundinacea* Schreb. plants treated with SeNPs at concentrations of 1.5, 3.0, and 4.5 mg/L. An increase in DPPH scavenging activity was reported, reaching 284.26±1.81 mg Ascorbic Acid Equivalent (AAE)/100 g at 4.5 mg/L. Similarly, a study evaluating the absorption, translocation, and effects of nZnS at concentrations of 0, 0.1, 0.5, and 1 mg L^−1^ in mung bean (*Vigna radiata*) plants reported increased DPPH scavenging activity in treated plants, with the highest activity observed at 0.1 mg L^−1^ nZnS. This underscores the significance of nanoparticle (NP) application doses.^[50]^

In our study, the lowest ABTS free radical scavenging activity was recorded with a 41.66 % increase in the A_1_ and A_2_ applications, and a similar 44 % increase in the SA_1_ application compared to salt treatment. Salt treatment reduced the ABTS free radical scavenging activity in the control group by 108.33 %. Additionally, the CUPRAC copper (II) ion reducing power results indicated the highest increase of 76.52 % in the A3 application compared to the control group, and 103.98 % in the SA_3_ application compared to salt treatment. FRAP iron (III) ion reducing power recorded the highest increase of 59.50 % in the A_3_ application and 81.77 % in the SA_3_ application compared to salt treatment. In their 2017 study, Javed et al.^[51]^ investigated the effects of ZnO‐NP treatments on the phenolic content of drought‐stressed *Stevia rebaudiana* plants. They reported that ZnO‐NPs at concentrations of 100 and 1000 mg/L significantly reduced the phenolic content. However, they also noted that NP‐treated plants exhibited a marked increase in total phenolic compounds and non‐enzymatic activities (DPPH, ABTS, and FRAP), which mitigated lipid peroxidation and oxidative damage.[^52^, ^53^] Similar and contrasting results have been observed in our study.

## 4 Conclusions

The data obtained revealed the detrimental effects of saline conditions on pepper plants (*Capsicum annuum* L.). Salt application resulted in elevated MDA and proline levels in the plants. However, the application of selenium nanoparticles was observed to alleviate these increases, signifying a mitigation of salinity‐induced damage. Furthermore, a decline in the phenolic content of the plants, along with reduced DPPH and ABTS scavenging activities, was identified. Decreases in FRAP and CUPRAC reducing power activities were also noted. These findings suggest that selenium applications diminished physiological and biochemical stress in salt‐stressed plants compared to non‐treated ones. Moreover, the study highlighted differential effects of three distinct nanoparticle concentrations in mitigating salinity stress in pepper plants. SC‐SeNP applications under saline conditions demonstrated the potential of these nanoparticles to enhance plant tolerance.

In conclusion, this study demonstrates that the foliar application of Se‐NPs on pepper leaves serves as an effective strategy to enhance salinity tolerance. Selenium treatments preserved photosynthetic pigments better than salt treatments, maintained optimal osmotic conditions, and reduced cellular damage, as evidenced by proline and MDA data. The use of SeNPs through foliar application is recommended for cultivating pepper plants in saline soils due to its practicality. However, further extensive and detailed studies are necessary to assess field‐level suitability and plant adaptability.

## Conflict of Interests

The authors declare no conflict of interest.

## Data Availability

The data that support the findings of this study are available from the corresponding author upon reasonable request.
